# Gender representation in anesthesiology research: a historical perspective from the Brazilian Journal of Anesthesiology

**DOI:** 10.1016/j.bjane.2024.844584

**Published:** 2025-01-03

**Authors:** Stefania Lacerda Garcia, Claudia Marquez Simões, Maria José Carvalho Carmona, Liana Maria Tôrres de Araújo Azi

**Affiliations:** a Universidade Salvador, Salvador, Bahia, Brazil; b Hospital das Clínicas da Faculdade de Medicina da Universidade de São Paulo (HC-FMUSP), Instituto do Câncer do Estado de São Paulo, Serviços Médicos de Anestesia, São Paulo, SP, Brazil; c Universidade de São Paulo (USP), São Paulo, SP, Brazil; d Faculdade de Medicina da Universidade Federal da Bahia (FMUFBA), Salvador, BA, Brazil

Dear Editor,

The percentage of women in medical schools and residency programs has steadily increased over recent years. The 2023 demographic projection of the Brazilian medical workforce reveals that women will be the majority among doctors in Brazil from 2024 onward.^1^ In anesthesiology, despite the growing percentage of women, this gender disparity persists, particularly in scientific production within the specialty. In 1951, the Revista Brasileira de Anestesiologia (RBA) was established as the official publication of the Brazilian Society of Anesthesiology and, in 2019, with the journal's internationalization process, the journal was renamed the Brazilian Journal of Anesthesiology (BJAN). Analyzing gender dynamics in publications is important for understanding women's representation in Brazilian scientific historical evolution. This study aims to identify the prevalence of women as first authors of BJAN articles since its first issue as RBA. Additionally, the study sought to investigate the relationship between gender and the type of study.

The gender of each author was determined through online platforms such as LinkedIn, Google Scholar, and institutional websites, supplemented by gender-identification applications (e.g., genderize.io, gender.api). Data extraction was independently conducted by two authors, with a third author consulted in cases of discrepancies to ensure the accuracy and reliability.

Data were analyzed, collecting variables such as article title, gender, first author's name, year of publication, study type, volume, and issue number of the journal. Articles classified as “Notice” were excluded from the analysis. Over the 72-years, there were changes in the classification for submission of scientific works. For categorization in the present study, the current author guidelines on the journal's website were used. “Editorial”, “Case Report”, “Letters to the Editor”, “Short Communication” were maintained, while Clinical Research, Original Study, Experimental Study, Original Investigation, Clinical Study, Scientific Article or Special Article were grouped under “Original Investigation”, Narrative Review, Systematic Review or Review Article were grouped under “Review Article” and Clinical Images or Contextualized Images were grouped under “Clinical Image”. As “Others”, Infographic, Educational Article, Diverse Article, Erratum, Obituary, Tribute, News, Miscellaneous, and Clinical Information were considered.

This retrospective analysis encompassed all articles published in the BJAN from its inception in 1951 until December 2023. Among the 5,374 articles included, 928 (17.26%) listed a female as the first author. In only 15 (0.27%) articles, it was not possible to determine the gender. A progressive increase in female first authorships was observed between 1951 (0) and 2023 (49), with a significant peak in 2022 (65). The most published study types were “Original research” ‒ 2644 (49.19%), “Letters to the Editor” ‒ 663 (12.33%) and “Review article” ‒ 597 (11.10%). Women published more in “Original Research” ‒ 473 (15.86%) and in “Others” ‒ 212 (20.30%). There was a difference in gender proportions by type of article, but both genders published in all types of studies according to the current division of the journal (Fig. 1). Line charts with trend lines were used to illustrate gender prevalence trends over time, while bar charts depicted the distribution of gender across different study types.

**Figure 1:**
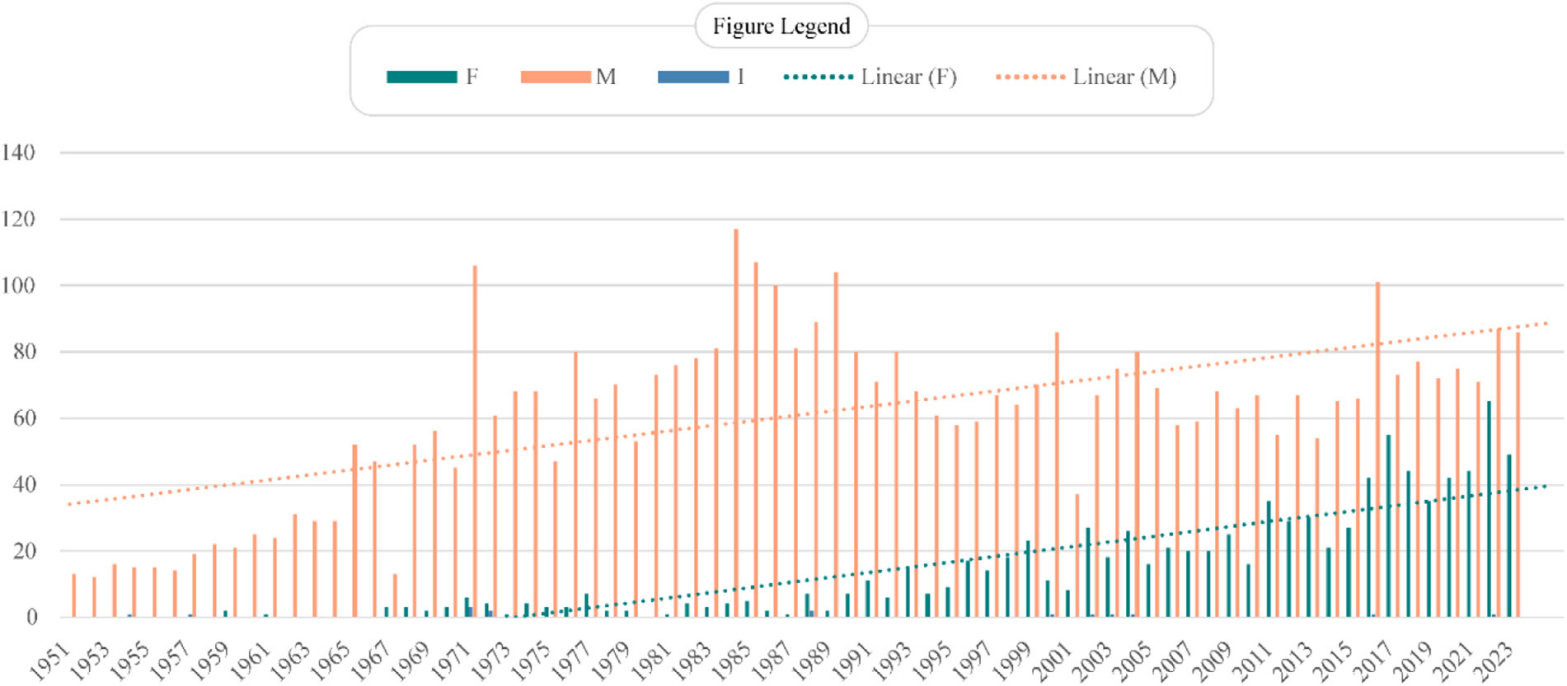
Trends in the number of male and female first authors in the Brazilian Journal of Anesthesiology (1951-2023). The figure displays the annual distribution of first authors by gender (M, Male; F, Female; I, Indefinite (when the gender could not be determined)) in the Brazilian Journal of Anesthesiology from 1951 to 2023. Bars represent the number of publications per year for male and female first authors, while dotted lines indicate linear trends for each gender.

Microsoft Excel (365,version 2406) was employed for data storage, analysis, and graphical presentation. Descriptive statistics summarized the data, offering insights into historical and temporal patterns of gender representation among authors. Chi-Square tests were applied to assess the association between gender and study type, and linear regression analysis was conducted to evaluate the primary outcome, providing a comprehensive understanding of the trends.

The linear regression analysis revealed that the proportion of female first authors increased by an average of 0.62% per year, with 72.8% of the variance explained by the model (R^2^ = 0.728). The correlation coefficient (R = 0.853) indicates a strong positive correlation between year and female authorship. By fitting regression lines for each gender and analyzing the point where these lines could potentially intersect, it was observed that the trends do not converge in the foreseeable future. This suggests that, although female participation is increasing, male publications continue to grow at a rate that maintains a relative gap. Additionally, a Chi-Square test was applied to evaluate the association between gender and type of study. The result was a p-value of 0, providing statistical evidence of an association between gender and type of study.

Since its inception, it took eight years for the first female authorship to appear in BJAN, marking a significant milestone in Brazilian anesthesiology. Since then, a proportional linear growth in female authorship has been observed, aligning with other authors that report a significant increase in female first authorship in top anesthesia journals over the past decade.^2^

Our analysis revealed an encouraging growth in female authorship, particularly notable in 2022 with 65 (42.48%) female first authorships. A study that evaluated the prevalence of women as first authors in three journals specializing in neuroanesthesiology and neurocritical care over the past five years found a peak in 2019, with a female representation of 37.45%.^3^ Similarly, the same study identified an increasing trend in female first authorship, rising from 20.5% of articles in 2002 to 30.2% in 2017, while in BJAN this proportion was higher, from 27.36% to 42.96% in the same period. Women's participation was notably high from 2016 to 2023, a period when two women largely occupied the role of Editor-in-Chief. This may have contributed to the increased participation of women, as they may have felt more represented and encouraged to submit their work.

Women appear to be gaining representation in anesthesiology publications, with recent studies reporting female representation as high as 42.8%.^4^^,^^5^ Many journals are actively diversifying their editorial boards by including more women in leadership roles such as editors-in-chief, associate editors, and reviewers. These initiatives can foster a more inclusive environment, potentially contributing to recent increases in female representation. To reduce the potential for gender bias, some journals have adopted double-blind review processes, where both the authors and reviewers are anonymous, a policy adopted also by BJAN.

The present study has several limitations. Firstly, the determination of the authors' gender was based on online searches and gender identification tools, which may have classification errors. To minimize these risks, gender assignment was verified by a second person and through artificial intelligence tools, with additional reviews made on specialized websites. In cases of discrepancies in the results (e.g., ambiguous or unclearly identified names), the classification was resolved by consensus, with selection from external sources, such as academic profiles or professional social networks. However, it is confirmed that, despite these validation efforts, methods based on online resources can be limited and introduce biases, such as inaccuracy in gender identification or lack of useful public information. Additionally, the analysis focused solely on first authorship, potentially overlooking significant contributions from women as co-authors or corresponding authors. Lastly, changes in editorial policies over the years may have influenced the categorization of study types, affecting the temporal comparability of the data.

Despite the limitations, this study makes a significant contribution by highlighting the evolving presence of women in scientific authorship within BJAN over seven decades. This study highlights positive trends in women's participation, reflecting advances in gender equity within Brazilian anesthesiology, similar to trends observed internationally. This growth reflects efforts and advances in gender equity within anesthesiology, although challenges remain. Continuing to monitor and support female participation in research is essential to achieving truly equitable and diverse representation in anesthesiology science.

## Authors’ contribution

Stefania Lacerda Garcia: This author contributed to Conceptualization; Investigation; Data Collection; Methodology; Project administration; Supervision, and Writing-original draft, review & editing; Claudia Marquez Simões: Supervision-review & editing; Maria José Carvalho Carmona: Supervision-review; Liana Maria Tôrres de Araújo Azi: This author contributed to Methodology; Formal analysis; Data curation; Project administration; Supervision and Writing-review & editing.

## Prior presentations

Work was approved for oral presentation at the Brazilian Congress of Anesthesiology (CBA).

## Conflicts of interest

The authors declare no conflicts of interest.
